# Metabolic Regulation of Hepatitis B Virus Infection in HBV-Transgenic Mice

**DOI:** 10.3390/metabo12040287

**Published:** 2022-03-25

**Authors:** Wenning Lan, Yang Wang, Zixiong Zhou, Xia Sun, Yun Zhang, Fangrong Zhang

**Affiliations:** 1 Key Laboratory of Gastrointestinal Cancer, Ministry of Education, School of Basic Medical Sciences, Fujian Medical University, Fuzhou 350122, China; wnl@mail.ustc.edu.cn; 2 Xiamen Key Laboratory of Rare Earth Photoelectric Functional Materials, Xiamen Institute of Rare Earth Materials, Haixi Institute, Chinese Academy of Sciences, Xiamen 361021, China; 3 Ganjiang Innovation Academy, Chinese Academy of Sciences, Ganzhou 341001, China; 4 Institute of Immunotherapy, Fujian Medical University, Fuzhou 350122, China; wangyang@fjmu.edu.cn; 5 Department of Pathology, School of Basic Medical Sciences, Fujian Medical University, Fuzhou 350122, China; zzxpathology@fjmu.edu.cn; 6 Fujian Science and Technology Innovation Laboratory for Optoelectronic Information of China, Fuzhou 350108, China; sunxia@fjoel.cn; 7 State Key Laboratory of Structural Chemistry, Fujian Institute of Research on the Structure of Matter, Chinese Academy of Sciences, Fuzhou 350002, China; 8 Fujian Key Laboratory of Tumor Microbiology, Department of Medical Microbiology, Fujian Medical University, Fuzhou 350122, China

**Keywords:** HBV infection, NMR spectroscopy, tissues, metabolomics

## Abstract

Hepatitis B virus (HBV) infection is a worldwide health burden. Metabolomics analysis has revealed HBV-induced metabolism dysregulation in liver tissues and hepatocytes. However, as an infectious disease, the tissue-specific landscape of metabolic profiles of HBV infection remains unclear. To fill this gap, we applied untargeted nuclear magnetic resonance (NMR) metabolomic analysis of the heart, liver, spleen, lung, kidney, pancreas, and intestine (duodenum, jejunum, ileum) in HBV-transgenic mice and their wild-type littermates. Strikingly, we found systemic metabolic alterations induced by HBV in liver and extrahepatic organs. Significant changes in metabolites have been observed in most tissues of HBV-transgenic mice, except for ileum. The metabolic changes may provide novel therapeutic targets for the treatment of HBV infection. Moreover, tissue-specific metabolic profiles could speed up the study of HBV induced systemic metabolic reprogramming, which could help follow the progression of HBV infection and explain the underlying pathogenesis.

## 1. Introduction

HBV, a hepatotropic DNA virus, is the pathogen of acute and chronic hepatitis B in humans. HBV infection is one of the most severe public health security problems globally. Primary liver cancer mainly caused by HBV was the sixth most commonly diagnosed cancer and the third leading cause of cancer death worldwide in 2020, with approximately 906,000 new cases and 830,000 deaths [1]. To date, the primary treatments for HBV infection include antiviral, anti-inflammatory, antioxidant, anti-fibrosis, immune regulation, and symptomatic therapies [2,3,4,5,6]. Currently, no drugs or effective treatment can be utilized for the complete clearance of HBV, which means that treatment must continue for life [7]. HBV reinfection has become a major risk factor for prognosis, especially HBV infection in extrahepatic organs [8,9]. Understanding the pathogenesis of HBV will bring new insights into the potential therapies.

The emergence of metabolomics studies has accelerated the better understanding of HBV infection pathogenesis and treatment. Metabolomics uncovers the metabolic profiles of an isolated organ, cell, tissue, biological fluid, or organ connections [10]. Nuclear magnetic resonance (NMR) spectroscopy has been extensively used for metabolomics studies [11]. NMR-based metabolomics can provide an untargeted, quantitative snapshot of global metabolite abundance [12], while it is less time-consuming in detecting a number of metabolites and sample preparation, highly reproducible, non-destructive, and non-invasive compared to mass spectrometry (MS) [13]. Currently, dysregulated metabolism of HBV infection on host cells has been revealed, including lipid, vitamin, glucose, amino acid, bile acid, and nucleic acid metabolism [14,15,16,17,18]. However, previous studies have provided some insights into the role of HBV-induced metabolism dysregulation in liver tissues and hepatocytes, while other organs that may be affected by HBV have been neglected [19,20]. Viral hijacking is usually systemic, not just one organ [21,22]. HBV cDNA has been observed in serum, kidneys, skeletal muscle, pancreas, even in hair and fingernails, and not only the liver [23,24,25]. The progression of HBV is notoriously difficult to follow in whole organisms. Metabolomics is an emerging but powerful tool for following the systemic metabolic reprogramming of HBV infection to provide biological information.

Metabolic regulation is an adaptive mechanism by which proliferating cells adapt to their increasing energy demands and nutrient-poor microenvironment [26,27]. This nutrient-poor microenvironment is a highly heterogeneous ecosystem composed of cells and subcells with interactive signals, low oxygen, and pH, leading to abnormal metabolism of sugar, pyruvate, amino acids, and lipids, thus promoting tumor formation, invasion, and metastasis [26,28]. HBV infection triggers metabolic regulation, inflammation and/or fibrosis processes, with the production and activation of cytokines, chemokines, and growth factors, as well as leukocyte infiltration, tissue immunity, and fibroblast population activation in the whole organism, which is considered to conduce to the various stages of HBV-HCC (hepatocellular carcinoma) development [29]. As a crucial predisposing factor in HCC, HBV can coordinate and control liver metabolism by binding transcription factors, thus driving systemic metabolic reprogramming [30]. For example, activation of PPARα associated with fatty acid oxidation is required for rapid induction of HBV transcription [31]. Taken together, molecular dysfunctions triggered by viral infection are throughout the organism, where different organs are connected by blood and lymph vessels. For instance, in the end-stage of HBV infection, bilirubin that cannot degrade in the liver will reach the brain and alter its function, resulting in hepatic encephalopathy (HE) [32,33]. A recent study has shown that the HBV-related microenvironment is more immunosuppressive and exhausted than the nonviral-related microenvironment [34]. Our previous data show that HBV-HCC undergoes a global metabolic reprogramming during tumor growth, which occurs not only in tumors but also in peritumor tissue [12]. As tumors progress from premalignant lesions to locally invasive tumors to metastatic cancers, metabolic phenotypes evolve increasingly clearly [35]. Therefore, further studies of virus-induced metabolic reprogramming, especially in the different organs and premalignant lesions, could help to explain the underlying pathogenesis and how it invades and metastasizes. 

In this study, we found HBV-induced metabolic reprogramming does not only occur in the liver, but that there were significant metabolic differences in the heart, spleen, kidneys, and parts of the small intestine. To this end, we applied untargeted NMR spectroscopy and obtained substantial changes in metabolites of the heart, liver, spleen, lung, kidney, pancreas, and intestine from HBV-transgenic mice compared to their wild-type littermates. We observed changes of metabolites in all detected tissues and highlighted their potential influence on metabolic reprogramming. In addition, we identified a series of metabolites, such as branched-chain amino acids (BCAAs, commonly known as valine, isoleucine and leucine), a general potential biomarker in HBV-transgenic mice, there was an increasing trend in most tissues, and choline could be a liver-specific biomarker for HBV detection. Taken together, a more profound comprehension of the potential processes might provide new sights on the HBV infection pathologies and assist in finding potential targets of the therapies.

## 2. Results

### 2.1. Multivariate Analysis of ^1^H NMR Spectroscopic Data Revealed Significant Differences in Tissues Metabolic Profiles between HBV-Transgenic and Control Mice

**Figure 1:**
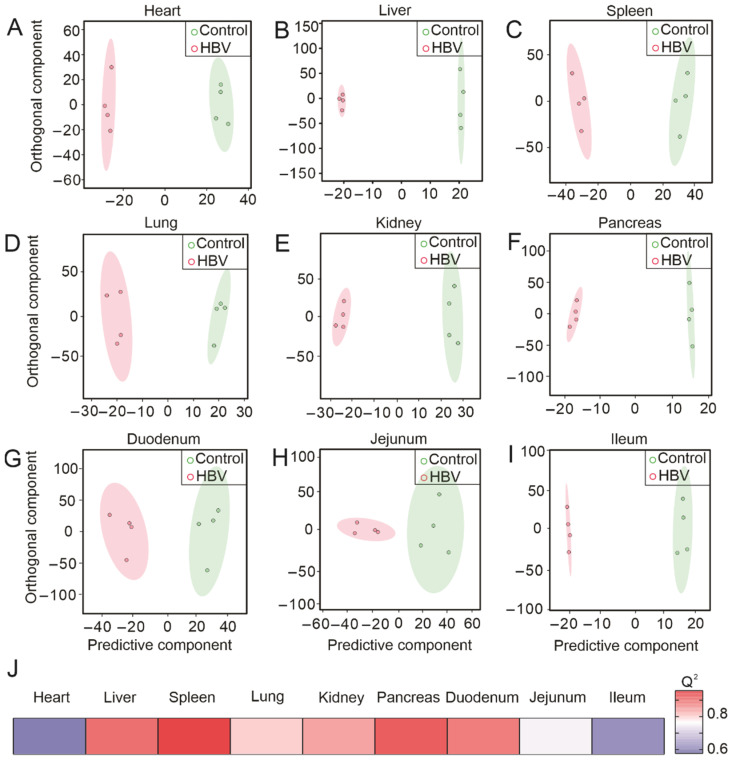
Multivariate analysis of ^1^H NMR spectroscopic data revealed significant differences in tissues metabolic profiles between HBV-transgenic and control mice. (**A**–**I**) represents the O-PLS-DA plot of the heart, liver, spleen, lung, kidney, pancreas, duodenum, jejunum, and ileum, respectively. (**J**) Heatmap showing O-PLS-DA-derived Q^2^ for pairwise comparisons of the different organs samples.

Given that HBV infection is a systemic disease [21], we hypothesized that the tissues-specific metabolic landscape of HBV-transgenic mice had undergone significant changes compared with that of control mice. The NMR profiles of the heart samples between the HBV-transgenic and control mice showed a significant difference in the orthogonal-partial least squares-discriminant analysis (O-PLS-DA) plot (Figure 1A). The permutation test provided the correlation coefficients R^2^Y up to 0.996 (*p* = 0.21) and a positive Q^2^ of 0.576 (*p* = 0.25) (Figure S1A). Then, comparing the metabolic profiles between liver samples isolated from HBV-transgenic and control mice, they were distinct in the OPLS-DA (Figure 1B), with correlation coefficients R^2^Y of up to 0.999 (*p* = 0.16) and a Q^2^ of 0.905 (*p* = 0.03) (Figure S2A), further indicating the significance of these results. The samples of spleen between the HBV-transgenic and control mice revealed a significant difference in the O-PLS-DA plot (Figure 1C), while the correlation coefficients Q^2^ of up to 0.957 (*p* = 0.08) and a R^2^Y of 0.992 (*p* = 0.08) (Figure S3A) in the permutation test were verified. The O-PLSDA models discriminated NMR spectra of lung samples from HBV-transgenic and control mice (Figure 1D). The permutation test provided the correlation coefficients Q^2^ of up to 0.801 (*p* = 0.04) and a R^2^Y of 0.991 (*p* = 0.08) (Figure S4A). The O-PLS-DA score plots (Figure 1E) of kidneys presented a clear distinction between samples with correlation coefficients Q^2^ of up to 0.844 (*p* = 0.03) and a positive R^2^Y of 0.996 (*p* = 0.04) (Figure S5A). Notably, the metabolites spectra of pancreas samples from HBV-transgenic and control mice were separated clearly in the O-PLS-DA score plots (Figure 1F), with correlation coefficients Q^2^ of up to 0.927 (*p* = 0.03) and a positive R^2^Y of 0.998 (*p* = 0.06) (Figure S6A). In small intestinal system, we found that the metabolites of duodenum samples between HBV-transgenic and control mice separated in the O-PLS-DA score plots (Figure 1G), and the correlation coefficients of Q^2^ was up to 0.888 (*p* = 0.04) and a positive R^2^Y of 0.966 (*p* = 0.09) (Figure S7A). The metabolites spectra of jejunum samples were distinguishable in the O-PLS-DA score plots (Figure 1H) with the correlation coefficients Q^2^ of up to 0.75 (*p* = 0.04) and a positive R^2^Y of 0.92 (*p* = 0.09) (Figure S8A). Unexpectedly, compared to other samples, the metabolites of ileum samples showed a slight difference in the O-PLS-DA score plots (Figure 1I), with a correlation coefficients Q^2^ of up to 0.602 (*p* = 0.5) and a positive R^2^Y of 0.998 (*p* = 0.75) (Figure S9A). HBV-transgenic mice underwent a systemic metabolic reprogramming, which was particularly significant in the liver, spleen, kidney, pancreas, duodenum, and liver (Figure 1J). 

### 2.2. Tissue-Specific Metabolomic Fingerprints and Related Metabolic Pathways

**Figure 2:**
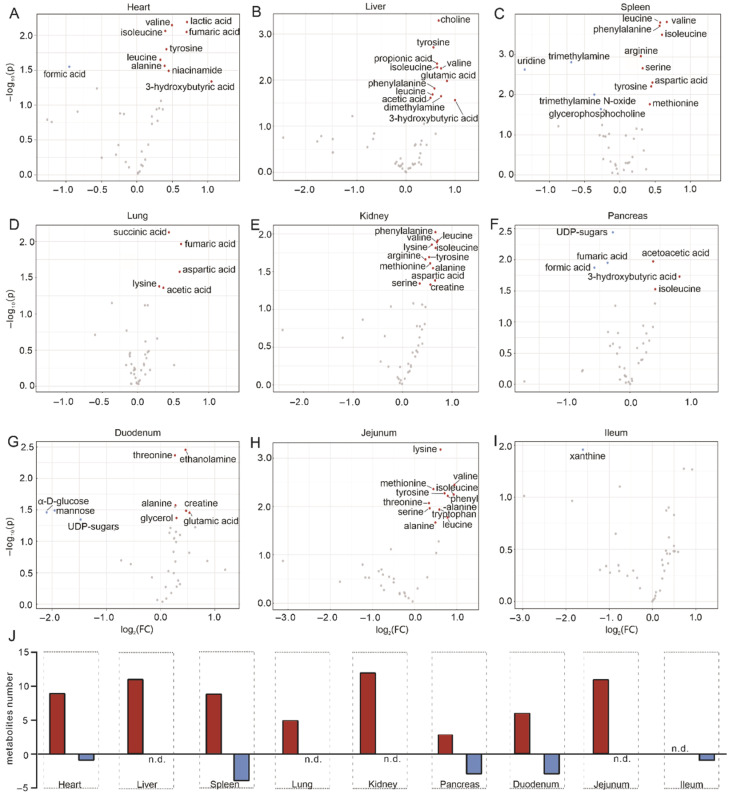
Metabolite changes in different tissues. (**A**–**I**) represents the Volcano plot of the heart, liver, spleen, lung, kidney, pancreas, duodenum, jejunum, and ileum, respectively. Increased (red) and decreased (blue) metabolites illustrate significant fold changes during HBV infection, while grey dots represent insignificantly changed metabolites. (**J**) Summary of the changed metabolites number from the volcano plot in different tissues, red indicates an increased number, and blue indicates a decreased number of metabolites (n.d., not detectable).

**Figure 3:**
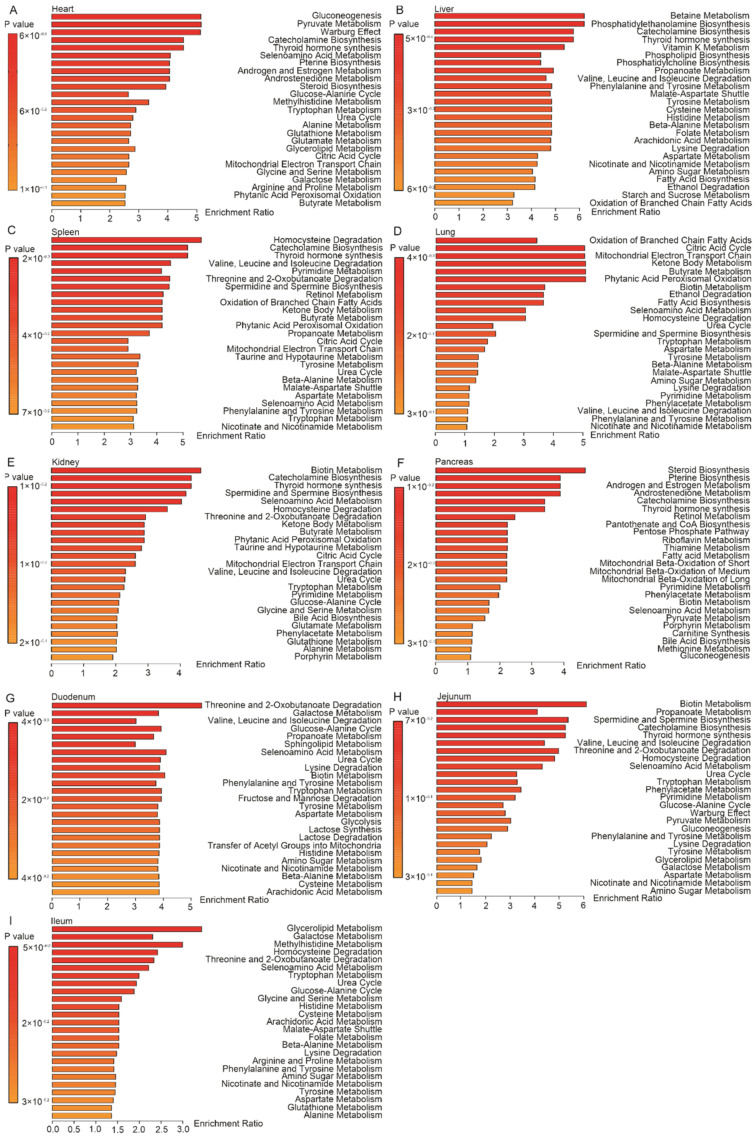
MSEA of affected metabolites highlighting their physiological relevance. (**A**–**I**) represents the heart, liver, spleen, lung, kidney, pancreas, duodenum, jejunum, and ileum, respectively.

Extrahepatic manifestations of HBV infection have been observed and some of them have been underestimated for a long time. For instance, HBV-related cirrhosis may face cardiac dysfunction as a complication, which includes volume restrictions and decreased systemic vascular resistance [36]. In line, we observed metabolic changes in the hearts of HBV-transgenic mice compared to control mice. The volcano plot and reduced spectra of metabolites in the heart displayed the altered metabolites, including the increased lactic acid, valine, isoleucine, fumaric acid, tyrosine, leucine, alanine, niacinamide, and 3-hydroxybutyric acid, while formic acid decreased (Figure 2A and Figure S1B,C). Metabolite enrichment analysis (MSEA), an analysis method, revealed the relationships between the metabolic pathways and altered metabolites, which showed that the changes in metabolites of heart were associated with gluconeogenesis, and pyruvate metabolism, Warburg effect, catecholamine biosynthesis, and thyroid hormone synthesis metabolic pathway (Figure 3A). Additionally, with the liver being the critical organ in metabolism and nutritional regulation, it is no wonder that HBV infection has been shown to induce metabolic disorders of the organism [37,38]. In the volcano plot and reduced spectra of liver samples, many metabolites increased, such as choline, tyrosine, propionic acid, isoleucine, valine, l-phenylalanine, glutamic acid, leucine, dimethylamine, acetic acid, and 3-hydroxybutyric acid (Figure 2B and Figure S2B,C). According to the MESA, betaine metabolism, phosphatidylethanolamine biosynthesis, catecholamine biosynthesis, thyroid hormone synthesis, and vitamin K metabolism were related to the changes in liver samples (Figure 3B). Moreover, the invasion of HBV also causes immune responses in the organism [39], and the spleen is the largest peripheral lymphatic organ in the body, so it will definitely be affected by the immune response [40]. In our study, we obtained the metabolites that were altered clearly from the volcano plot—leucine, valine, phenylalanine, isoleucine, arginine, serine, aspartic acid, tyrosine, and methionine were the rising group, while uridine, trimethylamine, trimethylamine N-oxide, and glycerophosphocholine were the descending group in spleen samples (Figure 2C and Figure S3A,B). In the MESA, the significantly altered metabolites in the spleen were mainly related to homocysteine degradation, catecholamine biosynthesis, thyroid hormone synthesis, leucine, isoleucine degradation, and pyrimidine metabolism (Figure 3C). According to the previous studies, long-term exposure to HBV increases the risk of asthma [41] and is associated with a poorer prognosis of non-small cell lung cancer [42]. Lung metastasis significantly decreases the survival of HBV-HCC [43]. The volcano plot and reduced spectra of metabolites in the lung indicated that succinic acid, fumaric acid, l-aspartic acid, l-lysine, and acetic acid all increased (Figure 2D and Figure S4B,C). The MESA revealed that the metabolites of lung samples are relevant to the oxidation of branched-chain fatty acids, citric acid cycle, mitochondrial electron transport chain, ketone body metabolism, and butyrate metabolism (Figure 3D).

More than that, a direct association of HBV infection with kidney diseases [44,45,46,47,48], especially with glomerular diseases [49,50], has already been demonstrated. HBV DNA has been detected in kidneys of HBV-related glomerulonephritis [51]. Phenylalanine, valine, leucine, lysine, isoleucine, arginine, tyrosine, methionine, alanine, aspartic acid, serine, and creatine increased, which could be obtained in the volcano plot and reduced spectra of kidney samples (Figure 2E and Figure S5B,C). The MESA supplied the pathways that influenced kidney samples’ metabolites: biotin metabolism, catecholamine biosynthesis, thyroid hormone synthesis, spermidine and spermine biosynthesis, and selenoamino acid metabolism (Figure 3E). At present, HBV infection increases the risk of pancreatic cancer, as confirmed in many studies [52,53,54]. The volcano plot and reduced spectra showed increased acetoacetic acid, 3-hydroxybutyric acid, and isoleucine. In the meantime, decreases in uridine diphosphate-sugars (UDP-sugars), fumaric acid, and formic acid were observed in the pancreas (Figure 2F and Figure S6B,C).

In the MESA, we found that the metabolites of pancreas involved pathways were steroid biosynthesis, pterine biosynthesis, androgen and estrogen metabolism, androstenedione metabolism, and catecholamine biosynthesis (Figure 3F). Because of the presence of the gut–liver axis, HBV has been shown to induce intestinal dysbiosis [55]. To complete our systemic study of HBV-infection in metabolically active tissue, we reported the metabolic changes in the duodenum, jejunum, and ileum of HBV transgenic and control mice. The information on metabolites changes was from the volcano plot and reduced spectra, which indicated that threonine, ethanolamine, alanine, creatine, glycerol, and glutamic acid were increased, in contrast, to mannose, α-D glucose, and UDP-sugars that were deduced in the duodenum (Figure 2G and Figure S7B,C). The MESA of the duodenum supplied the pathways, including the threonine and 2-oxobutanoate degradation, galactose metabolism, valine, leucine, and isoleucine degradation, glucose-alanine cycle, and propanoate metabolism (Figure 3G). Increased metabolites in jejunum included lysine, valine, methionine, tyrosine, isoleucine, threonine, phenylalanine, serine, tryptophan, leucine, and alanine (Figure 2H and Figure S8B,C). Among them in jejunum were related to the biotin metabolism, propanoate metabolism, spermidine and spermine biosynthesis, catecholamine biosynthesis, and thyroid hormone synthesis, which was provided in the MESA (Figure 3H). Unexpectedly, the volcano plot and reduced spectra of ileum presented only one metabolite, xanthine (Figure 2I and Figure S9B,C). The metabolic pathway of ileum involved glycerolipid metabolism, galactose metabolism, methylhistidine metabolism, homocysteine degradation, threonine, and 2-oxobutanoate degradation shown in the MESA (Figure 3I). Significant changes in metabolites were observed in most tissues of HBV-transgenic mice, except for the ileum (Figure 2J). 

### 2.3. Overview

**Figure 4:**
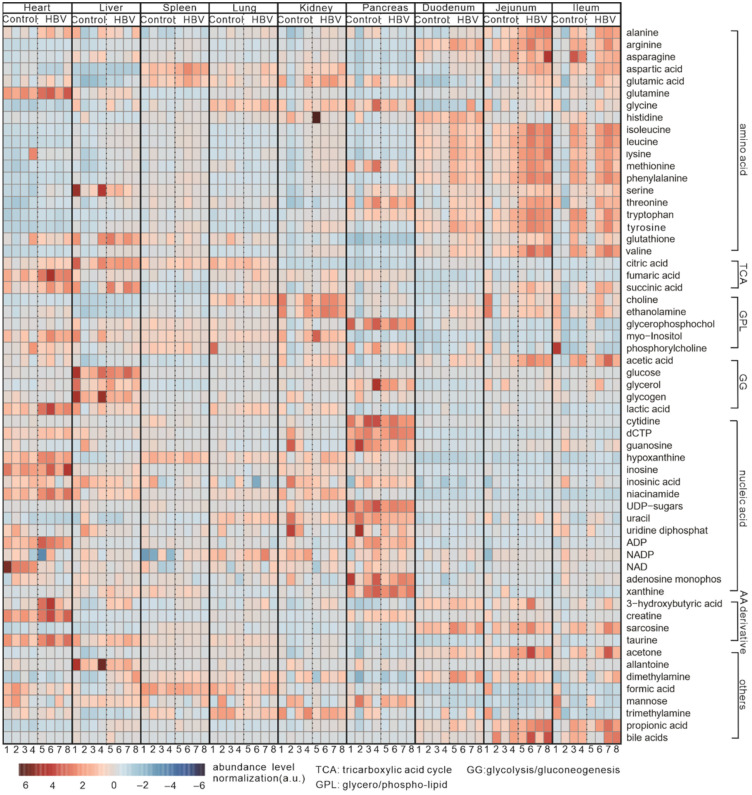
Heatmap of NMR analyses showing the relative metabolite levels in organs from HBV infected and control mice. The number of samples is divided into 1-8 in each group. A single sample was represented in columns, while relative metabolites were represented in rows. Red and blue show the increase and decrease, respectively. Metabolites are indicated and sorted according to different chemical classes or bio-molecular pathways.

We summarized all changes in the metabolites of the nine different tissues between HBV-transgenic and control mice, and the results are shown in Figure 4. This heatmap highlights the distribution of metabolites and related metabolic processes of HBV-transgenic mice, primarily including the enrichment of amino acids in the kidney and intestine, and the increase of the metabolites that related to the tricarboxylic acid cycle (TCA) and glycolysis/gluconeogenesis (GG) in the heart, liver, spleen, lung, and pancreas.

## 3. Discussion

HBV, a “metabolic virus”, affects many metabolic processes [18]. Our study revealed that metabolic reprogramming induced by HBV infection occurs not only in the liver but also in extrahepatic organs, such as the heart, liver, spleen, lung, kidney, pancreas, and intestine. A few studies have discussed the role of HBV infection on the risk of cardiovascular diseases and have shown the relationship between the two remains controversial [56]. Available evidence supports that HBV infection does not increase relative cardiovascular disease risk but is associated with metabolic syndrome [57,58]. However, HBV carriers with an increased prevalence of carotid atherosclerosis have been revealed by Ishizaka et al. [59]. We found that the metabolic profiles of heart were significantly changed in HBV transgenic mice. Compared to control mice, lactic acid and fumaric acid accumulated the most in the heart of HBV-transgenic mice, which may be due to the myocardial ischemia and hypoxia resulting in a blocked tricarboxylic acid cycle [60]. Previous studies pointed out the incidence of heart failure [61], and other cardiovascular diseases [62] are positively correlated with levels of BCAAs. In line, we observed increased levels of BCAAs in heart lysates of HBV-transgenic mice, suggesting a higher risk of heart disease after HBV infection. Moreover, we observed increased alanine, niacinamide, and 3-hydroxybutyric acid, the intermediate products of energy metabolism, further suggesting that the energy metabolism of the heart was affected by HBV replication [17]. In future clinical practice, whether HBV patients are more prone to cardiovascular dysfunction and the relationship between cardiovascular dysfunction and BCAAs metabolism deserves further study. 

The metabolic profiles of liver samples from HBV-infected mice are notably different from those from control mice. The altered metabolites induced by HBV infection were involved in the choline, amino acid, carbohydrates, and nucleotides metabolism, in line with a previous study [17]. In line with our results, we found elevated concentrations of choline in HBV-transgenic mice [63], which may be related to the biofilm synthesis and lipid metabolism to provide phosphatidylcholine for HBV replication [17]. Moreover, the increase in tyrosine and glutamic acid accelerated the synthesis of proteins to meet the protein requirements for HBV transcription [64]. In response to the oxidative stress caused by HBV replication, the concentration of BCAAs increases to produce more alternative antioxidants under GSH deficiency [65,66]. Taken together, the increase in the process of metabolism could be considered an obvious characteristic during the progression of liver diseases. The metabolic features of HBV infection in liver could shed new light on the pathological mechanism and provide potential therapeutic targets. 

Fourteen changed metabolites have been shown in the spleen of HBV-transgenic mice. Among them, metabolites are involved in metabolic pathways such as glucose, amino acid, and lipid metabolism. BCAAs promote glycolysis by stimulating glucose uptake to support metabolic reprogramming of immune cells [67,68]. In line with this observation, the high level of BCAAs may represent an increased immune response of the spleen induced by HBV infection. In addition, a previous study demonstrated that serine, as an essential immuno-metabolite, directly regulates adaptive immunity by controlling the capacity of T cell proliferation [69]. The elevated serine concentration in our data implied HBV infection may change T cell expansion. Accordingly, phenylalanine, tyrosine, and methionine as key elements for protein synthesis [70,71], and the increase in their concentrations suggested that HBV accelerates protein synthesis in the spleen [40]. Moreover, uridine deficiency can reduce the ability of spleen cells to secrete interleukin-2, reduce the activity of natural killer cells, and even affect the humoral immune response to T cell-dependent antigens [72]. In detail, we observed that the level of uridine was reduced in samples, which may result from impaired spleen function. Strikingly, we observed large changes in metabolites associated with immune response, which highlights inherent regulatory mechanisms of metabolic profiles benefiting HBV-induced invasion and metastasis.

In the lungs, a panel of 5 metabolites of HBV-transgenic mice was presented and related mostly to amino-acid metabolism (aspartic acid and lysine), glycolysis (acetic acid), and the TCA cycle (succinic acid and fumaric acid). Previous studies have reported that HBV protein and nucleic acids have been detected in the lung tissues [73], and exposure to HBV also increases the risk of lung disease [41]. According to our data, HBV infection does affect the metabolism of the lung, although the difference is not as significant as the liver. In general, energy expenditure in the lungs is classified as performing usual cellular tasks such as gene transcription and protein translation [74]. Accelerated processes such as the pulmonary tricarboxylic acid cycle and glycolysis may indicate that the lungs provide the energy for HBV replication. In line with elevated levels of succinic acid, an inflammatory signaling molecule suggested that the lungs may become inflamed due to HBV infection [75,76]. Lysine levels are associated with inflammation [77], and the increase of lysine indicated that there may be inflammation in the lung of HBV-transgenic mice. The lungs of HBV-transgenic mice showed changes in metabolites associated with inflammation, which may reveal how HBV affects lung function through metabolic reprogramming.

Damage to the kidney from HBV does not usually result from direct infection. In fact, the abnormal reaction to the virus of the immune system may contribute more to disease causation. In the kidney, we observed profound changes in metabolic spectra, with more than 12 metabolites differing between HBV-transgenic and control mice, which are mostly linked by amino-acid metabolism. Oral supplement of amino acids has been reported to arrest progressional renal failure in uremic patients, which suggests that amino acids may be involved in the repair of kidney damage [78]. Protective effects of alanine, aspartic acid, and arginine against renal injury and failure have been revealed [78,79]. Similarly, increased amino acid concentrations in the kidneys of HBV-transgenic mice may be in response to some HBV-induced kidney injury. Arginine is the precursor of creatine, and there is the possibility of synchronization change between them. As mentioned above, BCAAs and lysine can enhance the immunity of the organism, and the increase of BCAAs in our data indicated that HBV infection may alter the immune defense of the kidney. In this regard, Blachier et al. found that methionine directly regulates the immune system with increased production of taurine, glutathione, and other metabolites [80], and we found a similar increase in methionine in the kidneys of HBV-transgenic mice. In addition, the kidney is involved in the synthesis and exchange in inter-organs of several amino acids [81], containing arginine, phenylalanine, and tyrosine [82,83]. The high level of these amino acids concentration indicates metabolic disorders in the kidneys of HBV-transgenic mice. 

From the metabolite profiles in pancreatic samples, the increase of acetoacetic acid and 3-hydroxybutyric acid was most striking, which involved glycolysis. Our results show that the increase of acetoacetic acid and 3-hydroxybutyric acid are in line with a study that metabolites changes of the pancreatic intraepithelial neoplasia (PanIN) mice, a precancerous lesion of pancreatic cancer, suggesting that similar metabolic changes occurred in the pancreas of HBV-transgenic mice. The abnormal changes of isoleucine in HBV-transgenic mice may be due to the dysfunction of tumor suppressor genes of P53, SIRT3, SIRT6, and the oncogenes of Ras, PI3K, Akt, and c-Myc [84]. Acetoacetic acid, 3-hydroxybutyric acid, and isoleucine might be used in as potential biomarkers to determine the status of the pancreas in HBV-transgenic mice. 

In the intestine, the metabolic processes of the duodenum and jejunum are affected by HBV infection while the ileum is almost unchanged. To date, studies have focused on the association of HBV and gut microbiota, while few metabolic analyses of intestinal tissue has been performed. The data on healthy intestinal tissue in the context of HBV infection are almost blank. Therefore, our experimental results could lay a foundation for further investigations, focusing on the changes of intestinal metabolism and related metabolites, as well as the potential causes or consequences. Amino acids have been demonstrated to play a key role in the health of the intestine, such as glutamic acid and methionine being able to maintain intestinal function [85], threonine being able to nourish microflora in the intestine [86], methionine’s ability to improve the ability of intestinal antioxidants [87], and arginine and threonine’s ability to improve intestinal immune function [88]. In the duodenum, the levels of threonine, alanine, glutamic acid, and ethanolamine increased significantly in HBV-transgenic mice, implying that the intestinal function may change in HBV-transgenic mice. In the jejunum, metabolic changes were similar to those in the duodenum, but notably increased levels of BCAAs, serine, and lysine, which were related to immunity. Previous studies have reported that the intestine used BCAAs as fuel to renew its defense system against harmful pathogens [68]. This may indicate a similar immune response in different tissues of HBV transgenic mice.

## 4. Materials and Methods

### 4.1. Animals and Diets

For all experiments, organs isolated from age-matched female (6 to 8 weeks old) HBV-transgenic mice and their wild-type littermates (C57BL/6J) were used in this study [89] (*n* = 4). Mice were maintained in a clean, temperature-controlled (22 ± 1 °C) environment with light–dark cycle (12 h/12 h) regularly and free access to diet and water. Mice were fasted for 12 h before dissection. All experiments were performed in accordance with the Fujian Medical University Institutional Animal Care and Use Committee (IACUC). The mouse model of HBV was successfully established and evaluated in a previous study [89,90]. Microscopic examination was performed on the livers from HBV-transgenic and control mice. As shown in Figure S10, HBV-infected mice showed increased inflammation and cell edema (Figure S10B) compared to control group (Figure S10A).

### 4.2. NMR Sample Preparation

Organ samples were frozen rapidly in liquid nitrogen and stored at −80 °C until analysis. We resected 30–50 mg of each organ for the NMR metabolomics. To extract metabolites, we transferred the samples to a tube (contains 1.4 mm ceramic spheres, MP Biomedicals LLC, Santa Ana, CA, USA), then added 400 µL of ice-cold methanol and 200 µL MilliQ H_2_O to each tube for homogenization by FastPrep-24 tissue homogenizer (MP Biomedicals LLC, Santa Ana, CA, USA). After centrifugation at 13,000 rpm for 30 min (4 °C), the supernatant was transferred to a fresh tube and evaporated in a centrifugal evaporator (Eppendorf Concentrator plus, Hamburg, Germany) to generate a dry metabolite pellet. For the NMR experiments, samples were re-dissolved in 500 µL of NMR buffer (0.08 M Na_2_HPO_4_, 5 mM TMSP (3-(trimethylsilyl) propionic acid-2,2,3,3-d4 sodium salt), 0.04 (*w*/*v*) % NaN_3_ in D_2_O, pH adjusted to 7.4 with 8 M HCl and 5 M NaOH).

### 4.3. Data Acquisition

NMR measurements for ^1^H NMR metabolic profiling and analyses were performed as described and using a Bruker Avance III HD 600-MHz NMR spectrometer equipped with a TXI probe head. The Carr–Purcell–Meiboom–Gill (CPMG) pulse sequence was used to acquire ^1^H 1D NMR spectra with a pre-saturation for water suppression (cpmgpr1d, 512 scans, 73,728 points in F1, 12019.230 Hz spectral width, 1024 transients, recycle delay 4 s) (Figure S11A) [91,92]. NMR spectral data were processed as previously described [93,94]. Briefly, data were processed in Bruker Topspin version 4.0.2 using one-dimensional exponential window multiplication of the Free Induction Decay (FID), Fourier transformation, and phase correction. The NMR data were then imported into Matlab2014a; Trimethylsilyl propanoic acid (TSP) was used as the internal standard for chemical-shift referencing (set to 0 ppm); regions around the water, TSP and methanol signals were excluded; the NMR spectra were aligned; and a probabilistic quotient normalization (PQN) was performed for normalization to compensate for differences in concentration between samples [95]. Chenomx NMR suite 8.4 and reference compounds were used to identify the metabolites tissue during the analysis (Figure S11B). Approximately 60 metabolites above a signal-to-noise ratio (SNR) of 5 have been identified in mouse tissue extract samples. The integrations were used to generate the orthogonal partial least squares discriminant analysis (O-PLS-DA), permutation analysis, volcano plot, MSEA (including associated data consistency checks and cross-validation), and the heat map using MetaboAnalyst 5.0 [92]. The statistical significance of the identified differences was validated by the quality assessment statistic Q^2^.

### 4.4. Statistical Analysis

A univariate statistical analysis was carried out using GraphPad Prism (GraphPad Software, La Jolla, CA, USA). Data were represented as mean ± standard deviation (SD). The *p*-values were calculated using a two-tailed Student’s *t*-test for pairwise comparison of variables. Metabolites with *p* < 0.05 are shown in panel C of each supplementary figure [93]. Volcano plot with fold change >1.2, and *p*-value < 0.05.

### 4.5. Pathological Section Preparation

As described before [96], the collected liver tissues were fixed overnight in 10% formalin solution. Next, these tissues were processed with standard dehydration procedures and embedded in paraffin. Then, the embedded tissues were sectioned into 4-μm thick sections using a microtome (HM-340E, Thermo Fisher Scientific Inc., Waltham, MA, USA). These tissue sections were stained with hematoxylin and eosin (H&E) according to standard techniques and examined by a light microscope (BX53F, Olympus Corp., Tokyo, Japan).

## 5. Conclusions

A comprehensive metabolomic analysis of HBV infection in HBV-transgenic mice is provided by our current work. A limitation of our analysis concerns the small sample size (*n* = 4), which may increase the margin of error. HBV infection can induce metabolic reprograming in the organism, including liver and extrahepatic organs. The significantly increased choline in HBV-transgenic mice provides the potential to be a liver-specific biomarker. We distinguished valine, isoleucine, and leucine (BCAAs) to be altered in most tissues such as the heart, liver, spleen, kidney, and intestine, suggesting that they may be universal biomarkers in HBV-transgenic mice. BCAAs can be used as an energy supply during exercise [97] and as a fuel source by immune cells within the gut, which allows the immune system to regenerate itself more efficiently and protect against harmful pathogens [68]. This leads to the hypothesis that HBV infection mainly affects energy expenditure with increasing immune responses in most tissues. Lysine, an immunity-related amino acid [69,77], was observed to increase in the lung, kidney, and intestine, which suggested an increased probability of inflammatory responses in HBV-transgenic mice. Meanwhile, the concentration of serine was increased in the spleen, kidney, and intestine, providing potential evidence of inflammation in HBV-transgenic mice. Accordingly, HBV infection will lead to the patient being at higher risk of suffering kidney and intestinal disease. Therefore, the concentrations of lysine and serine in the kidneys and intestines can be the potential targets for diagnosis and intervention in the early stage of HBV infection. In addition, almost all of the tissues in HBV-transgenic mice showed changes in amino acid metabolism. Changes in the TCA cycle have been observed in the heart, liver, spleen, pancreas, and intestine of HBV-transgenic mice. Taken together, the tissue-specific metabolites discovered in the current study should provide a novel molecular read-out for HBV-associated changes of an organism. Further prospective studies are needed to demonstrate the applicability of these potential biomarkers in extrahepatic manifestations of HBV infected patient allocation for adjuvant trials and more frequent follow-up. Our results represent a powerful tool to follow the progression of HBV, especially in extrahepatic organs. Importantly, HBV hijacking is “hypermetabolic”. The systemic metabolic profiles of the tissues of HBV-transgenic mice provide an opportunity to understand the pathogenesis of HBV infection, invasion, and metastasis, and may help in developing powerful therapies.

## Data Availability

The data presented in this study are available on request from the corresponding author. The data are not publicly available due to the further study required.

## Supplementary Materials

The following supporting information can be downloaded at: https://www.mdpi.com/article/10.3390/metabo12040287/s1, Figure S1. (A) Cross validation (left) and permutation test with number *n* = 100 (right) from O-PLS-DA (Figure 1A, heart). (B) The reduced NMR spectrum revealed altered components in normalized heart samples. Positive covariance corresponds to components present at increased concentrations, whereas negative covariance corresponds to decreased component concentration. Predictivity of the model is represented by R^2^. 1 = leucine, 2 = isoleucine, 3 = valine, 4 = 3-hydroxybutyric acid, 5 = lactic acid, 6 = alanine, 7 = arginine, 8 = fumaric acid, 9 = tyrosine, 10 = formic acid, 11 = niacinamide. (C) Statistical analysis of altered metabolites in heart samples using a Student’s *t*-test. *p* < 0.05 was considered statistically significant. Figure S2. (A) Cross validation (left) and permutation test with number *n* = 100 (right) from O-PLS-DA (Figure 1B, liver). (B) The reduced NMR spectrum revealed altered components in normalized liver samples. Positive covariance corresponds to components present at increased concentrations, whereas negative covariance corresponds to decreased component concentration. Predictivity of the model is represented by R^2^. 1 = leucine, 2 = isoleucine, 3 = valine, 4 = propionic acid, 5 = 3-hydroxybutyric acid, 6 = acetic acid, 7 = glutamic acid, 8 = dimethylamine, 9 = choline, 10 = tyrosine, 11 = phenylalanine. (C) Statistical analysis of altered metabolites in heart samples using a Student’s *t*-test. *p* < 0.05 was considered statistically significant. Figure S3. (A) Cross validation (left) and permutation test with number *n* = 100 (right) from O-PLS-DA (Figure 1C, spleen). (B) The reduced NMR spectrum revealed altered components in normalized spleen samples. Positive covariance corresponds to components present at increased concentrations, whereas negative covariance corresponds to decreased component concentration. Predictivity of the model is represented by R^2^. 1 = isoleucine, 2 = valine, 3 = arginine, 4 = succinic acid, 5 = methionine, 6 = aspartic acid, 7 = trimethylamine, 8 = glycerophosphocholine, 9 = trimethylamine N-oxide, 10 = serine, 11 = threonine, 12 = tyrosine, 13 = phenylalanine, 14 = uridine. (C) Statistical analysis of altered metabolites in heart samples using a Student’s *t*-test. *p* < 0.05 was considered statistically significant. Figure S4. (A) Cross validation (left) and permutation test with number *n* = 100 (right) from O-PLS-DA (Figure 1D, lung). (B) The reduced NMR spectrum revealed altered components in normalized lung samples. Positive covariance corresponds to components present at increased concentrations, whereas negative covariance corresponds to decreased component concentration. Predictivity of the model is represented by R^2^. 1 = acetic acid, 2 = succinic acid, 3 = aspartic acid, 4 = lysine, 5 = fumaric acid. (C) Statistical analysis of altered metabolites in heart samples using a Student’s *t*-test. *p* < 0.05 was considered statistically significant. Figure S5. (A) Cross validation (left) and permutation test with number *n* = 100 (right) from O-PLS-DA (Figure 1E, kidney). (B) The reduced NMR spectrum revealed altered components in normalized kidney samples. Positive covariance corresponds to components present at increased concentrations, whereas negative covariance corresponds to decreased component concentration. Predictivity of the model is represented by R^2^. 1 = leucine, 2 = isoleucine, 3 = valine, 4 = alanine, 5 = arginine, 6 = methionine, 7 = aspartic acid, 8 = lysine, 9 = creatine, 10 = serine, 11 = tyrosine, 12 = phenylalanine. (C) Statistical analysis of altered metabolites in heart samples using a Student’s *t*-test. *p* < 0.05 was considered statistically significant. Figure S6. (A) Cross validation (left) and permutation test with number *n* = 100 (right) from O-PLS-DA (Figure 1F, pancreas). (B) The reduced NMR spectrum revealed altered components in normalized pancreas samples. Positive covariance corresponds to components present at increased concentrations, whereas negative covariance corresponds to decreased component concentration. Predictivity of the model is represented by R^2^. 1 = isoleucine, 2 = 3-hydroxybutyric acid, 3 = acetoacetic acid, 4 = UDP-sugars, 5 = fumaric acid, 6 = formic acid. (C) Statistical analysis of altered metabolites in heart samples using a Student’s *t*-test. *p* < 0.05 was considered statistically significant. Figure S7. (A) Cross validation (left) and permutation test with number *n* = 100 (right) from O-PLS-DA (Figure 1G, duodenum). (B) The reduced NMR spectrum revealed altered components in normalized duodenum samples. Positive covariance corresponds to components present at increased concentrations, whereas negative covariance corresponds to decreased component concentration. Predictivity of the model is represented by R^2^. 1 = alanine, 2 = glutamic acid, 3 = lysine, 4 = creatine, 5 = ethanolamine, 6 = glycerol, 7 = threonine, 8 = α-D glucose. (C) Statistical analysis of altered metabolites in heart samples using a Student’s *t*-test. *p* < 0.05 was considered statistically significant. Figure S8. (A) Cross validation (left) and permutation test with number *n* = 100 (right) from O-PLS-DA (Figure 1H, jejunum). (B) The reduced NMR spectrum revealed altered components in normalized jejunum samples. Positive covariance corresponds to components present at increased concentrations, whereas negative covariance corresponds to decreased component concentration. Predictivity of the model is represented by R^2^. 1 = leucine, 2 = isoleucine, 3 = valine, 4 = alanine, 5 = methionine, 6 = lysine, 7 = serine, 8 = threonine, 9 = tyrosine, 10 = phenylalanine, 11 = tryptophan. (C) Statistical analysis of altered metabolites in heart samples using a Student’s *t*-test. *p* < 0.05 was considered statistically significant. Figure S9. (A) Cross validation (left) and permutation test with number *n* = 100 (right) from O-PLS-DA (Figure 1I, ileum). (B) The reduced NMR spectrum revealed altered components in normalized ileum samples. Positive covariance corresponds to components present at increased concentrations, whereas negative covariance corresponds to decreased component concentration. Predictivity of the model is represented by R^2^. 1 = xanthine. (C) Statistical analysis of altered metabolites in heart samples using a Student’s *t*-test. *p* < 0.05 was considered statistically significant. Figure S10. Photomicrographs of representative sections of the livers from mice. Hematoxylin and eosin (H&E) staining of liver tissues was performed to assess liver injury. (A) Normal livers without HBV infection; (B) HBV-infected liver, showed significant increased infiltration of inflammatory cells (as denoted by the arrow in dark) and cell edema (as denoted by the arrow in white). Scale bars = 50 mm. Original magnification, X400.
