# Supplementary material for: Metabolic Regulation of Hepatitis B Virus Infection in HBV-Transgenic Mice
Source: Metabolites. 2022 Mar 25;12(4):287. doi: 10.3390/metabo12040287 (PMC9031567; doi:10.3390/metabo12040287)
Supplement: Supplementary file 1 [file metabolites-12-00287-s001.zip › metabolites-1640243-supplementary.pdf]

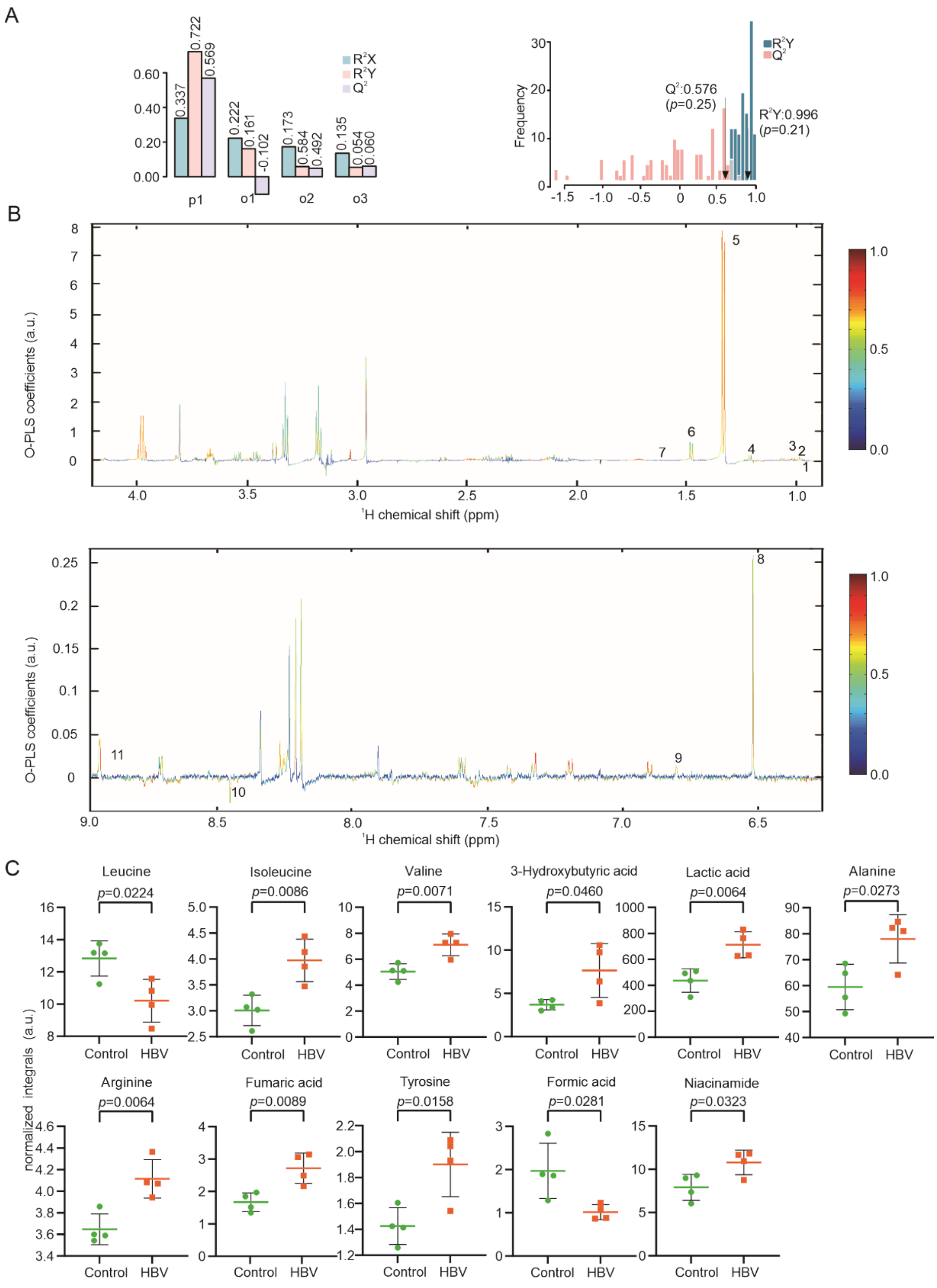

Supplementary Figure S1. (A) Cross validation (left) and permutation test with number n = 100 (right) from O-PLS-DA (Figure 1A, heart). (B) The reduced NMR spectrum revealed altered components in normalized heart samples. Positive covariance corresponds to components present at increased concentrations, whereas negative covariance corresponds to decreased component concentration. Predictivity of the model is represented by R^2. 1 = leucine, 2 = isoleucine, 3 = valine, 4 = 3-hydroxybutyric acid, 5 = lactic acid, 6 = alanine, 7 = arginine, 8 = fumaric acid, 9 = tyrosine, 10 = formic acid, 11 = niacinamide. (C) Statistical analysis of altered metabolites in heart samples using a Student's t-test. $p < 0.05$ was considered statistically significant.

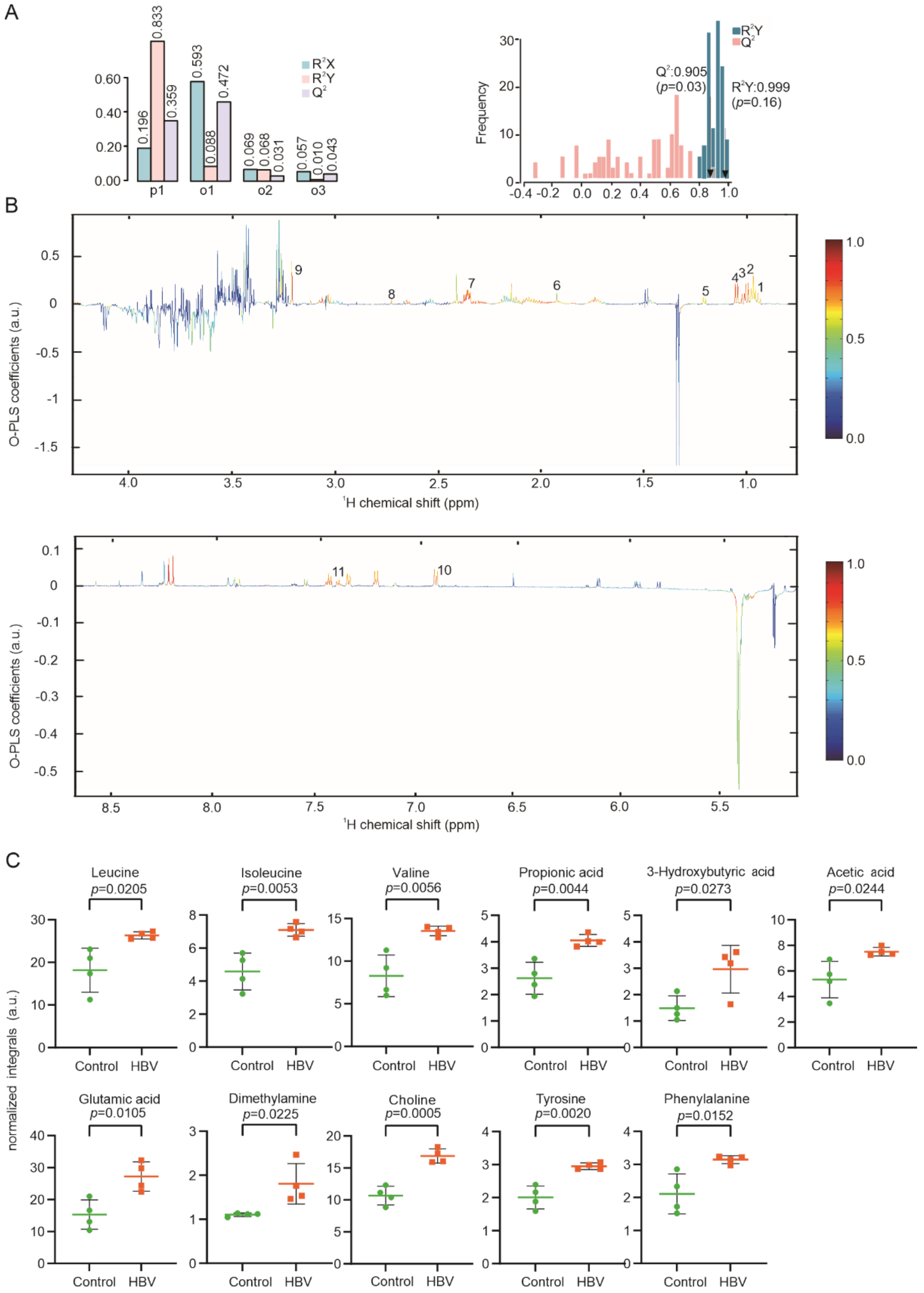

Supplementary Figure S2. (A) Cross validation (left) and permutation test with number n = 100 (right) from O-PLS-DA (Figure 1B, liver). (B) The reduced NMR spectrum revealed altered components in normalized liver samples. Positive covariance corresponds to components present at increased concentrations, whereas negative covariance corresponds to decreased component concentration. Predictivity of the model is represented by R^2. 1 = leucine, 2 = isoleucine, 3 = valine, 4 = propionic acid, 5 = 3-hydroxybutyric acid, 6 = acetic acid, 7 = glutamic acid, 8 = dimethylamine, 9 = choline, 10 = tyrosine, 11 = phenylalanine. (C) Statistical analysis of altered metabolites in heart samples using a Student's t-test. $p < 0.05$ was considered statistically significant.

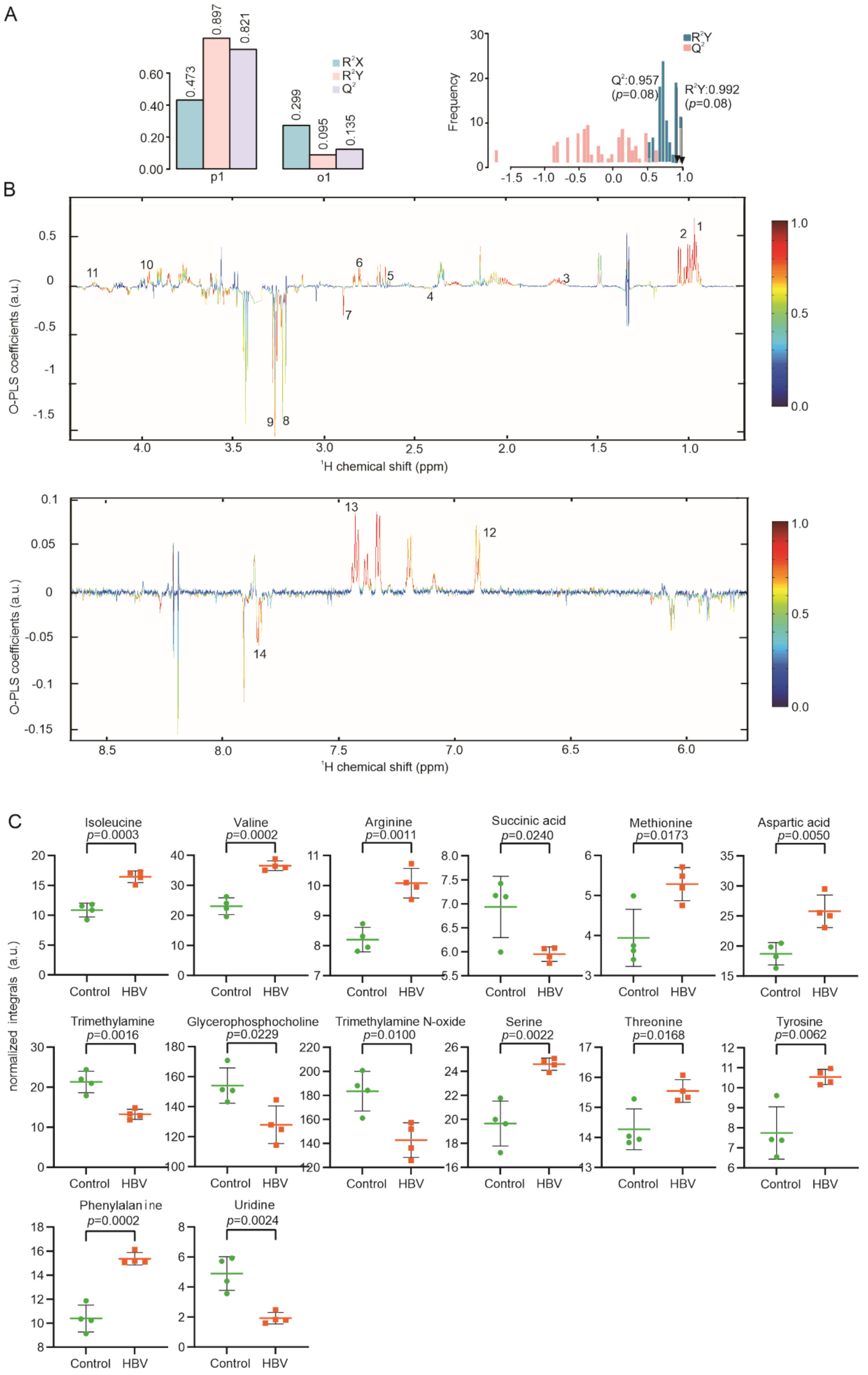

Supplementary Figure S3. (A) Cross validation (left) and permutation test with number n = 100 (right) from O-PLS-DA (Figure 1C, spleen). (B) The reduced NMR spectrum revealed altered components in normalized spleen samples. Positive covariance corresponds to components present at increased

concentrations, whereas negative covariance corresponds to decreased component concentration. Predictivity of the model is represented by R^2. 1 = isoleucine, 2 = valine, 3 = arginine, 4 = succinic acid, 5 = methionine, 6 = aspartic acid, 7 = trimethylamine, 8 = glycerophosphocholine, 9 = trimethylamine N-oxide, 10 = serine, 11 = threonine, 12 = tyrosine, 13 = phenylalanine, 14 = uridine. (C) Statistical analysis of altered metabolites in heart samples using a Student's t-test. $p < 0.05$ was considered statistically significant.

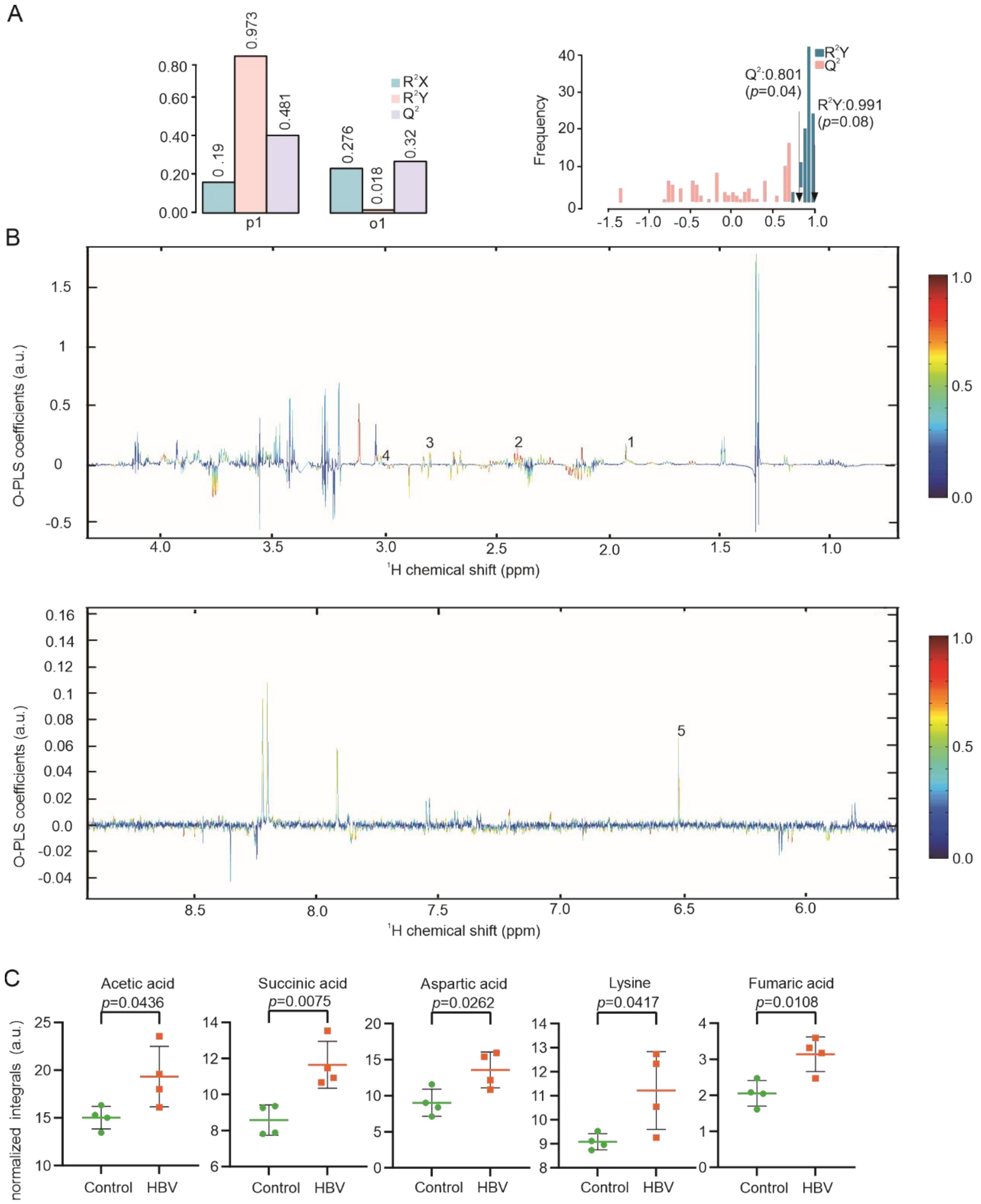

Supplementary Figure S4. (A) Cross validation (left) and permutation test with number n = 100 (right) from O-PLS-DA (Figure 1D, lung). (B) The reduced NMR spectrum revealed altered components in normalized lung samples. Positive covariance corresponds to components present at increased concentrations, whereas negative covariance corresponds to decreased component concentration. Predictivity of the model is represented by R^2. 1 = acetic acid, 2 = succinic acid, 3 = aspartic acid, 4 = lysine,

5 = fumaric acid. (C) Statistical analysis of altered metabolites in heart samples using a Student's t-test. $p < 0.05$ was considered statistically significant.

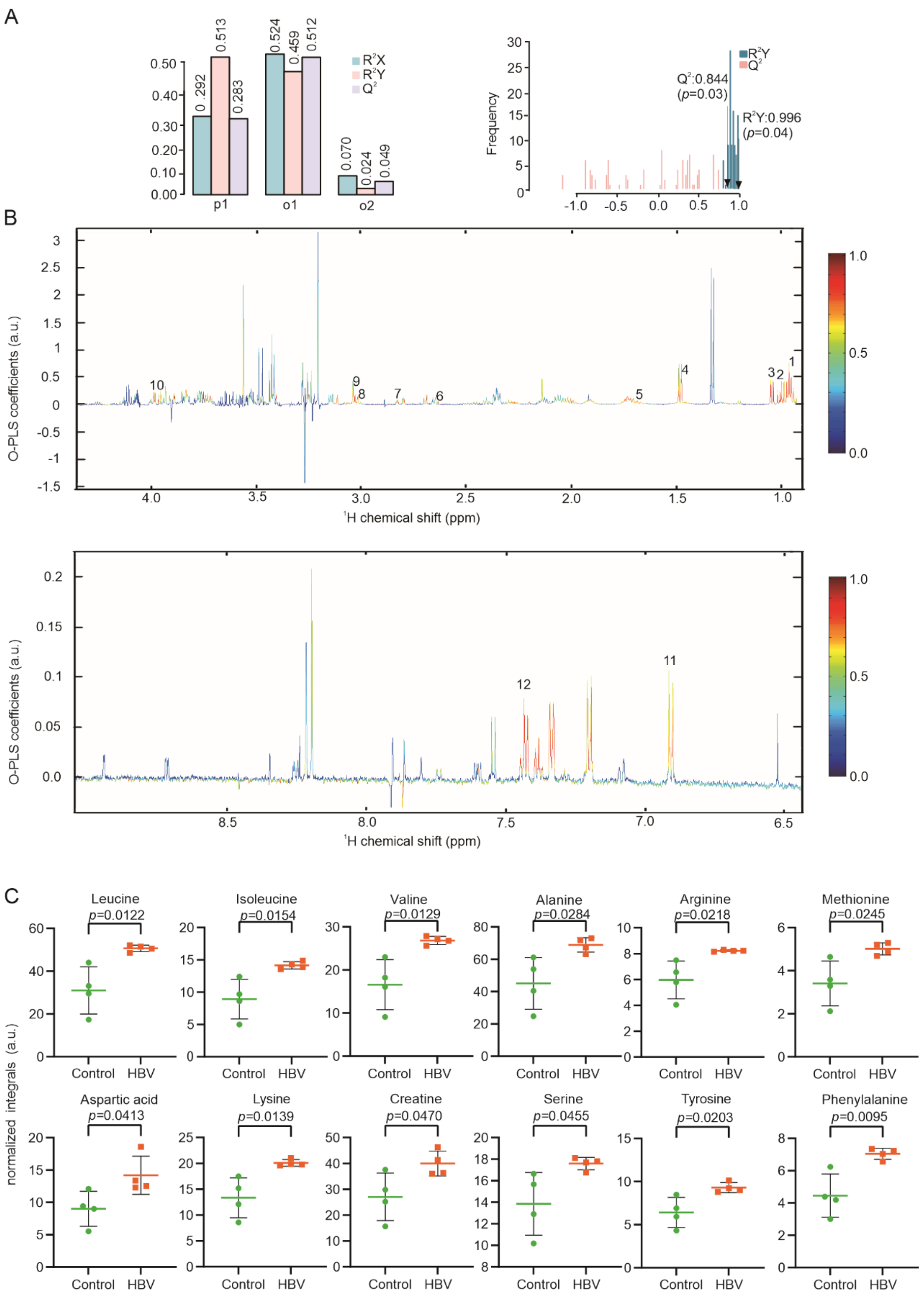

Supplementary Figure S5. (A) Cross validation (left) and permutation test with number n = 100 (right) from O-PLS-DA (Figure 1E, kidney). (B) The reduced NMR spectrum revealed altered components in normalized kidney samples. Positive covariance corresponds to components present at increased concentrations, whereas negative covariance corresponds to decreased component concentration. Predictivity of the model is represented by R^2. 1 = leucine, 2 = isoleucine, 3 = valine, 4 = alanine, 5 = arginine, 6 = methionine, 7 = aspartic acid, 8 = lysine, 9 = creatine, 10 = serine, 11 = tyrosine, 12 = phenylalanine. (C)

Statistical analysis of altered metabolites in heart samples using a Student's t-test. $p < 0.05$ was considered statistically significant.

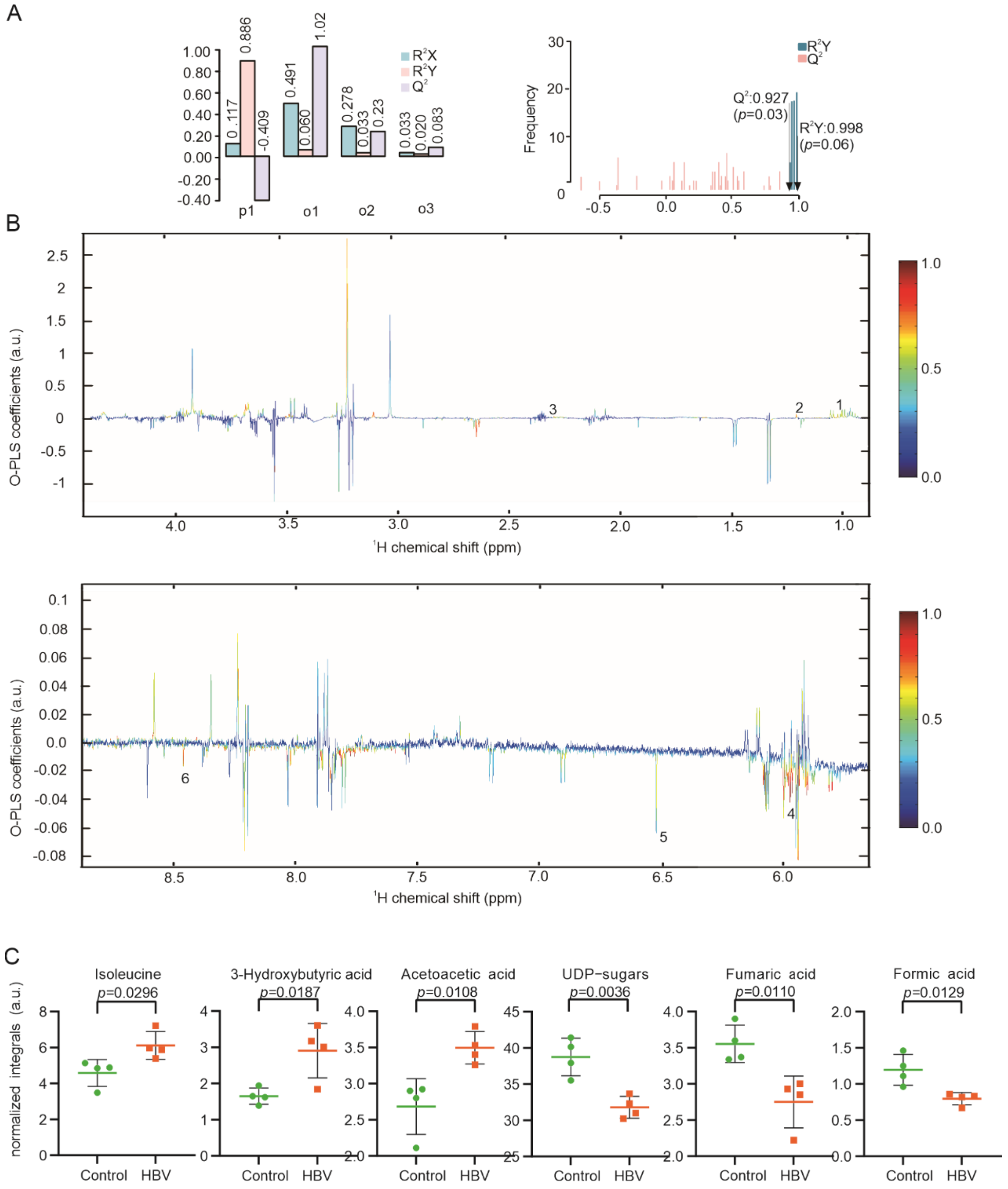

Supplementary Figure S6. (A) Cross validation (left) and permutation test with number n = 100 (right) from O-PLS-DA (Figure 1F, pancreas). (B) The reduced NMR spectrum revealed altered components in normalized pancreas samples. Positive covariance corresponds to components present at increased concentrations, whereas negative covariance corresponds to decreased component concentration. Predictivity of the model is represented by R^2. 1 = isoleucine, 2 = 3-hydroxybutyric acid, 3 = acetoacetic acid, 4 = UDP-sugars, 5 = fumaric acid, 6 = formic acid. (C) Statistical analysis of altered metabolites in heart samples using a Student's t-test. $p < 0.05$ was considered statistically significant.

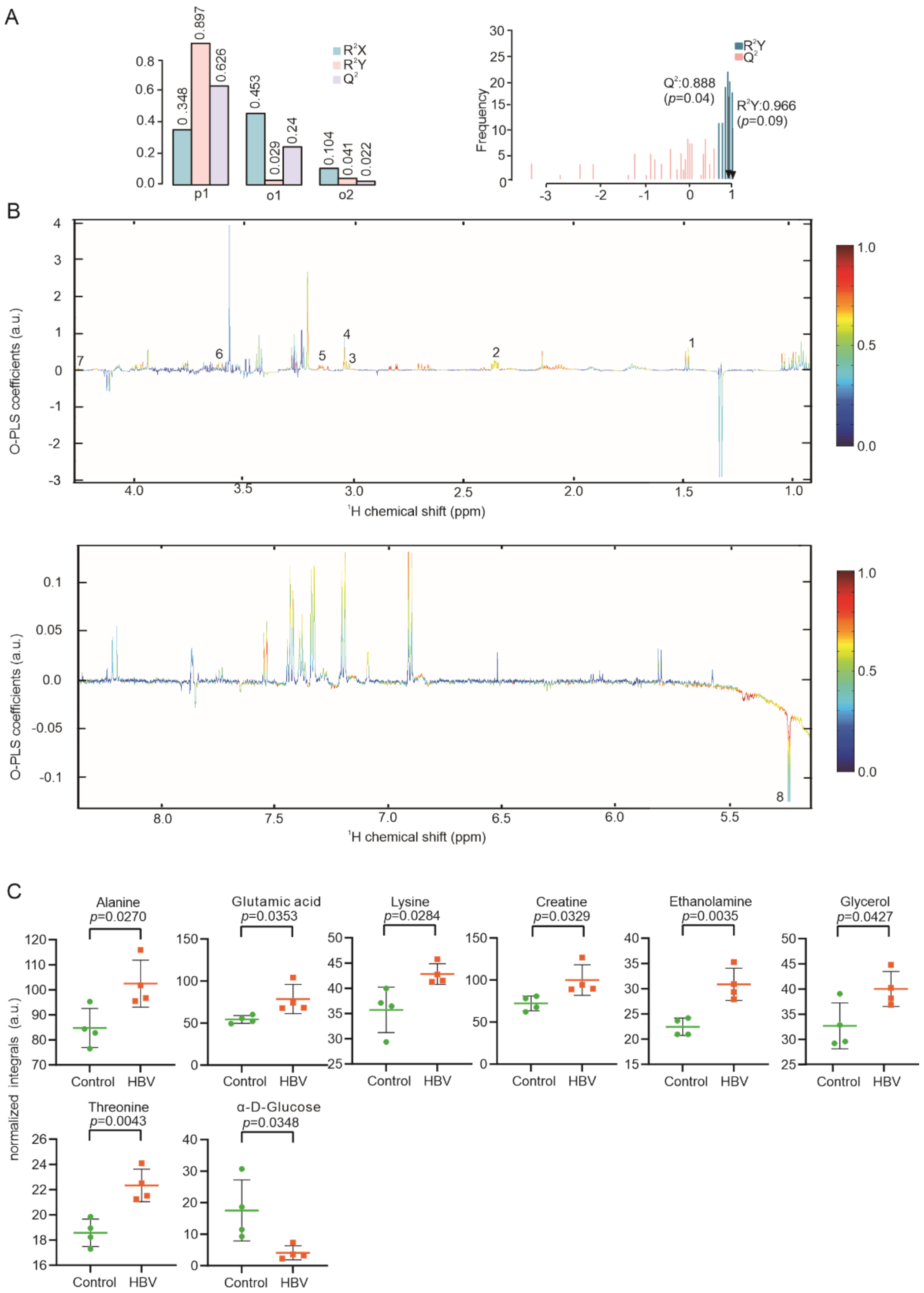

Supplementary Figure S7. (A) Cross validation (left) and permutation test with number n = 100 (right) from O-PLS-DA (Figure 1G, duodenum). (B) The reduced NMR spectrum revealed altered components in normalized duodenum samples. Positive covariance corresponds to components present at increased concentrations, whereas negative covariance corresponds to decreased component concentration. Predictivity of the model is represented by R^2. 1 = alanine, 2 = glutamic acid, 3 = lysine, 4 = creatine, 5 = ethanolamine, 6 = glycerol, 7 = threonine, 8 = α-D glucose. (C) Statistical analysis of altered metabolites in heart samples using a Student's t-test. $p < 0.05$ was considered statistically significant.

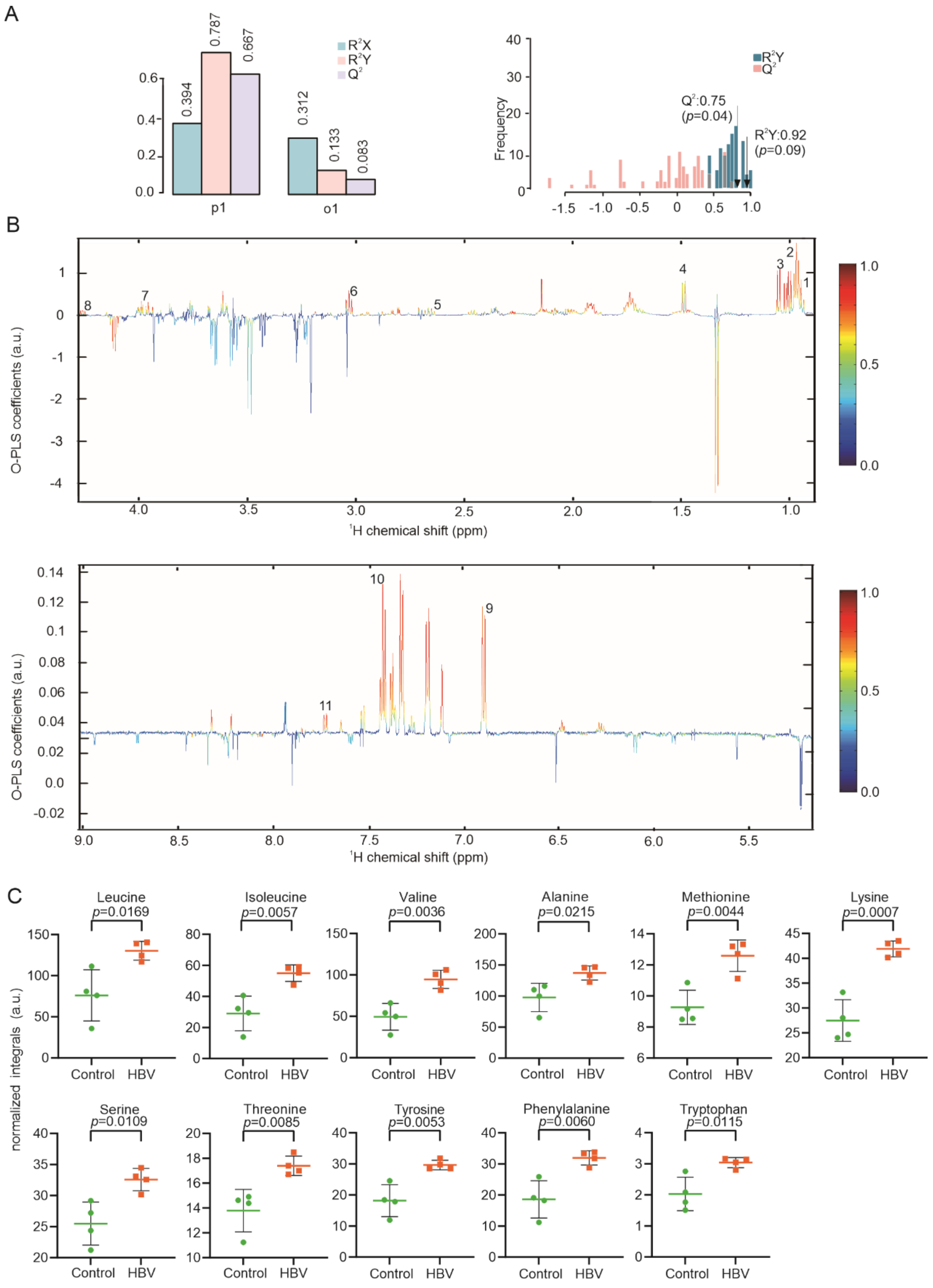

Supplementary Figure S8. (A) Cross validation (left) and permutation test with number n = 100 (right) from O-PLS-DA (Figure 1H, jejunum). (B) The reduced NMR spectrum revealed altered components in normalized jejunum samples. Positive covariance corresponds to components present at increased concentrations, whereas negative covariance corresponds to decreased component concentration. Predictivity of the model is represented by R^2. 1 = leucine, 2 = isoleucine, 3 = valine, 4 = alanine, 5 = methionine, 6 = lysine, 7 = serine, 8 = threonine, 9 = tyrosine, 10 = phenylalanine, 11 = tryptophan. (C) Statistical analysis of altered metabolites in heart samples using a Student's t-test. $p < 0.05$ was considered

statistically significant.

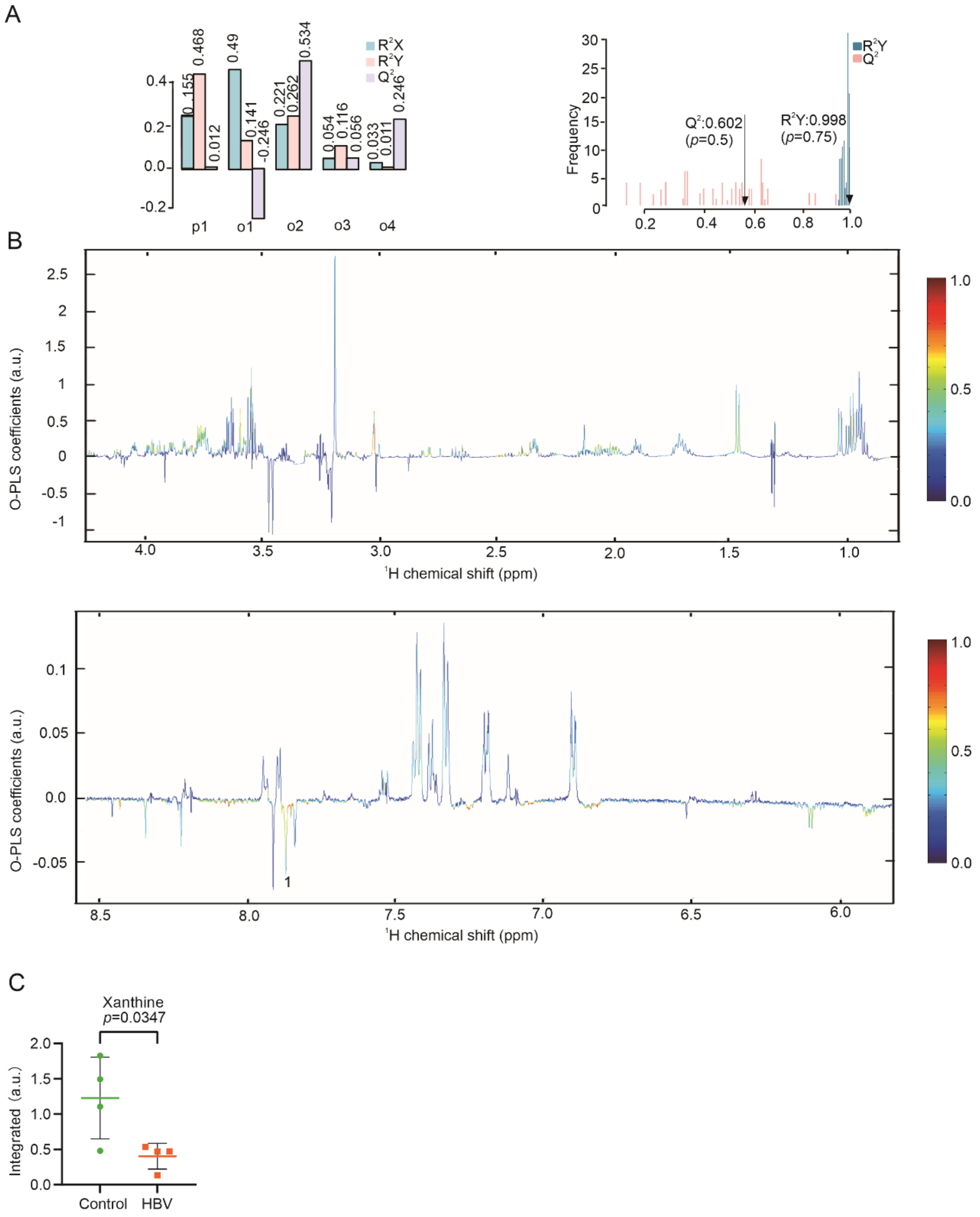

Supplementary Figure S9. (A) Cross validation (left) and permutation test with number n = 100 (right) from O-PLS-DA (Figure 1I, ileum). (B) The reduced NMR spectrum revealed altered components in normalized ileum samples. Positive covariance corresponds to components present at increased concentrations, whereas negative covariance corresponds to decreased component concentration. Predictivity of the model is represented by R^2. 1 = xanthine. (C) Statistical analysis of altered metabolites in heart samples using a Student's t-test. $p < 0.05$ was considered statistically significant.

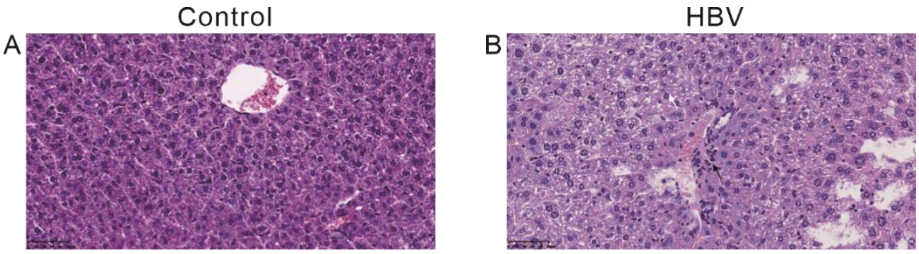

Supplementary Figure S10. Photomicrographs of representative sections of the livers from mice. Hematoxylin and eosin (H&E) staining of liver tissues was performed to assess liver injury. (A) Normal livers without HBV infection; (B) HBV-infected liver, showed significant increased infiltration of inflammatory cells (as denoted by the arrow in dark) and cell edema (as denoted by the arrow in white). Scale bars = 50 mm. Original magnification, X400.

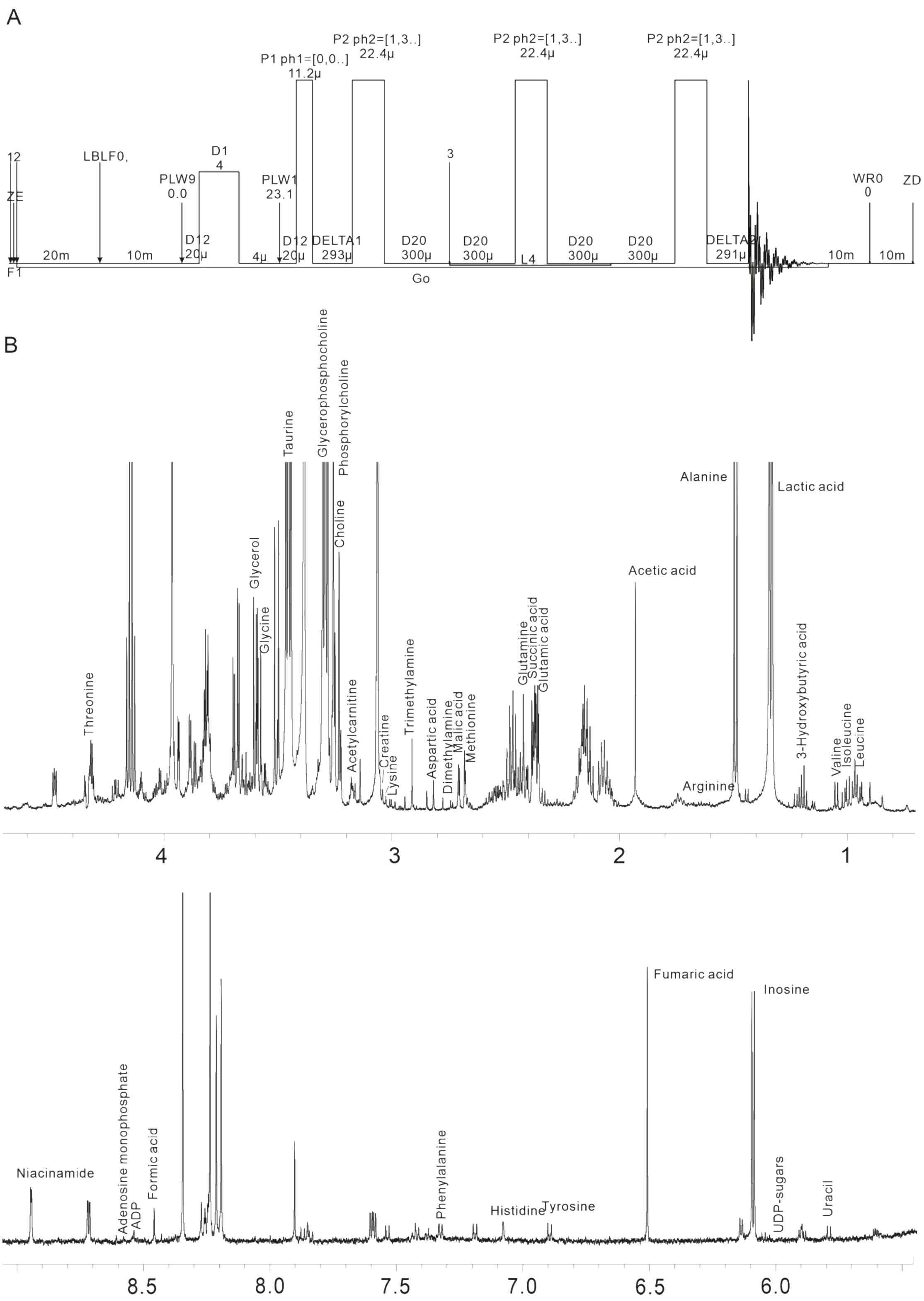

Supplementary Figure S11. (A) Pulse sequence of the 1D CPMG NMR experiment. (B)1D ^{1}H NMR example (control group, heart) spectrum with assigned metabolites.
